# Plasmon-Induced Transparency in an Asymmetric Bowtie Structure

**DOI:** 10.1186/s11671-019-3081-0

**Published:** 2019-07-23

**Authors:** Wei Wei, Xin Yan, Bing Shen, Xia Zhang

**Affiliations:** 1 School of Mechanical and Electric Engineering, Guangzhou University, Guangzhou, 510006 China; 2 Photonics Research Centre, Department of Electronic and Information Engineering, The Hong Kong Polytechnic University, Hung Hom, Kowloon, Hong Kong, China; 3 State Key Laboratory of Information Photonics and Optical Communications, Beijing University of Posts and Telecommunications, Beijing, 100876 China; 4 4Catalyzer Inc, Guilford, 06437 USA

**Keywords:** Surface plasmons, Plasmon-induced transparency, Electromagnetically induced transparency, Metal-insulator-metal

## Abstract

Plasmon-induced transparency is an efficient way to mimic electromagnetically induced transparency, which can eliminate the opaque effect of medium to the propagating electromagnetic wave. We proposed an aperture-side-coupled asymmetric bowtie structure to realize on-chip plasmon-induced transparency in optical communications band. The plasmon-induced transparency results from the strong coupling between the detuned bowtie triangular resonators. Either of the resonator works as a Fabry-Perot cavity with compact dimensions. The transparent peak wavelength can be easily controlled due to its strong linear relation with the resonator height. The ratio of absorption valley to the transparent peak can be more than 10 dB. Moreover, with excellent linearity of shifting wavelength to sensing material index, the device has great sensing performance and immunity to the structure deviations.

## Background

Electromagnetically induced transparency (EIT) effect, which results from quantum destructive interference between two pathways in three-level atomic systems [1, 2], shows tremendous potential applications in slow light propagation [3, 4], nonlinear optics [5], and optical storage [6]. In an EIT system, quantum interference effect reduces light absorption over a narrow spectral region, arising a sharp resonance of nearly perfect transmission within a broad absorption profile [7]. However, the EIT effect is very sensitive to broadening due to atomic motion. The realization of the EIT effect requires stable gas lasers and rigorous environments, which hampers its practical applications. Recently, kinds of configurations have been proposed to mimic EIT-like transmission without the demand of rigorous experimental conditions, including coupled micro-resonators [8–12], split-ring, and metamaterials [13–16] composed of dielectric and metal materials. Among them, metamaterial-based EIT with periodic unit patterns requires an excited signal light incident in the direction non-parallel to the chip surface. With the excited signal light incident in the direction parallel to the chip surface, coupled micro-resonators are remarkable to meet the requirement of on-chip integration applications of EIT-like transmission. To further reduce the footprint of EIT devices, plasmon-induced transparency (PIT) has been proposed as an analog to the classical EIT with strong optical confinement beyond the diffraction limit for electromagnetic waves [17–19]. Surface plasmons are optically induced oscillations of the free electrons at the interface of metal/dielectric exhibiting strong optical confinement and miniaturized photonic components [20, 21]. Recently, metal/insulator/metal (MIM) plasmonic waveguides with extremely high optical confinement and closer spacing to adjacent waveguides is a very promising nanoscale waveguide which is capable of overcoming the diffraction limit and has diverse applications of plasmonic sensors [22], couplers [23], and filters [24]. Thus, MIM-based PIT transmission has huge potential in on-chip applications of optical communications, optical information processing, and nonlinear optics.

Here, we propose a novel detuned resonators structure to obtain PIT transmission in MIM waveguides. The device with a planar structure is comprised of two detuned triangular resonators and one bus waveguide, forming asymmetric bowtie structure to enable PIT effect. Owing to the sensitive and linear response of transparent peak wavelength to structural parameters and medium inside the waveguide, the proposed device enables PIT-based refractive index sensing. With compact and easy-to-make structure, the device could be of great significance in on-chip photonic integrations.

## Methods

**Fig. 1:**
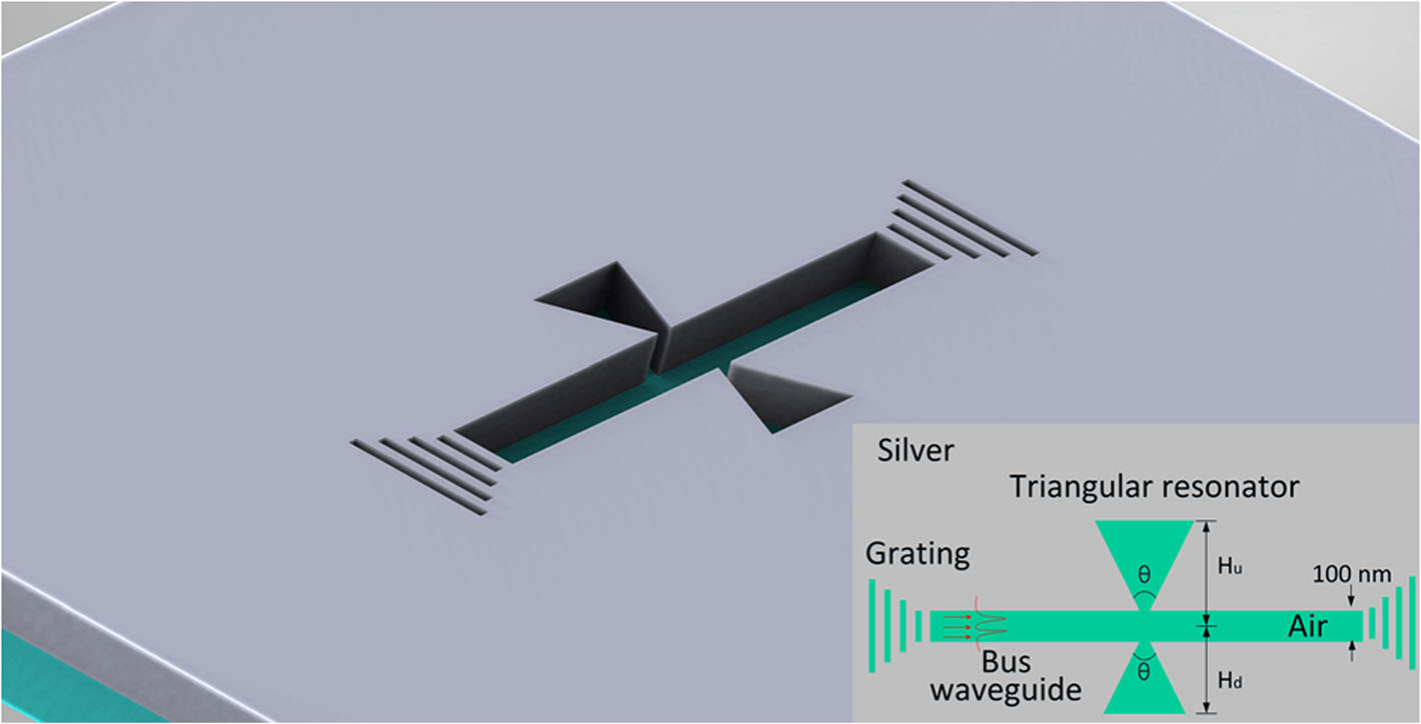
Schematic diagram of the asymmetric bowtie structure

The schematic of the asymmetric bowtie structure is depicted in Fig. 1, where the background material in blue is silver, whose permittivity is described by the Drude model of ${\varepsilon}_r={\varepsilon}_{\infty }-{\omega}_p^2/\left({\omega}^2+ j\gamma \omega \right)$, with *ε*_∞_=3.7, *ω*_*p*_=9.1 eV and *γ*=0.018 eV. The parameters adopted here in the above equation fit the experimental data at the optical communications frequencies [25]. All the MIM waveguides are filled by air. The long strip in the center of the structure is the bus waveguide for transmitting light. On both sides of the bus, waveguides are the bowtie resonators. The bowtie resonators are asymmetric with detuned structural parameters like altitude and angle being denoted by *H*_*u*_, *H*_*d*_, *θ*_*1*_, and *θ*_*2*_. The vortices of the triangles in the bowtie are in the middle of the bus waveguide. So, the bowtie resonators have small connections to the bus waveguide enabling efficient coupling between them. The width of the bus waveguides is fixed at 100 nm and the length of the bus waveguide has no effect on the PIT transmission spectrum except for transmission loss. So, its length is fixed at 1 μm considering the compactness and integration. Two gratings at both ends of the bus waveguide are to inject wide-band or wavelength-sweeping light source and collect transmission spectrum. Transmission spectrum was numerically calculated using the finite element method with scatter boundary conditions. In the numerical simulation, a plane wave was injected from the left grating of the bus waveguide by a port to excite fundamental TM modes of SPs. The transmitted light was collected from the right grating of the bus waveguide which is defined as *T* = *P*_out_/*P*_in_, where *P*_in_ =  ∫ *P*oavzd*S*_1_ and P_out_ =  ∫ Poavzd*S*_2_; *Poavz* is the *z* component of time-average power flow. The transmission spectra of the structure are obtained by parametrically sweeping the input wavelength. This asymmetric bowtie structure could be fabricated by steps as followings: first, deposit an Ag film with a thickness of 500 nm on a silica/silicon substrate; then, deposit a silica film with a thickness of 500 nm; last, fabricate the required pattern including gratings by EBL and etching. The proposed aperture-coupled scheme potentially has less stringent fabrication requirements than devices based on evanescent coupling and can be used to achieve efficient coupling in other important MIM plasmonic structures.

## Results and Discussion

**Fig. 2:**
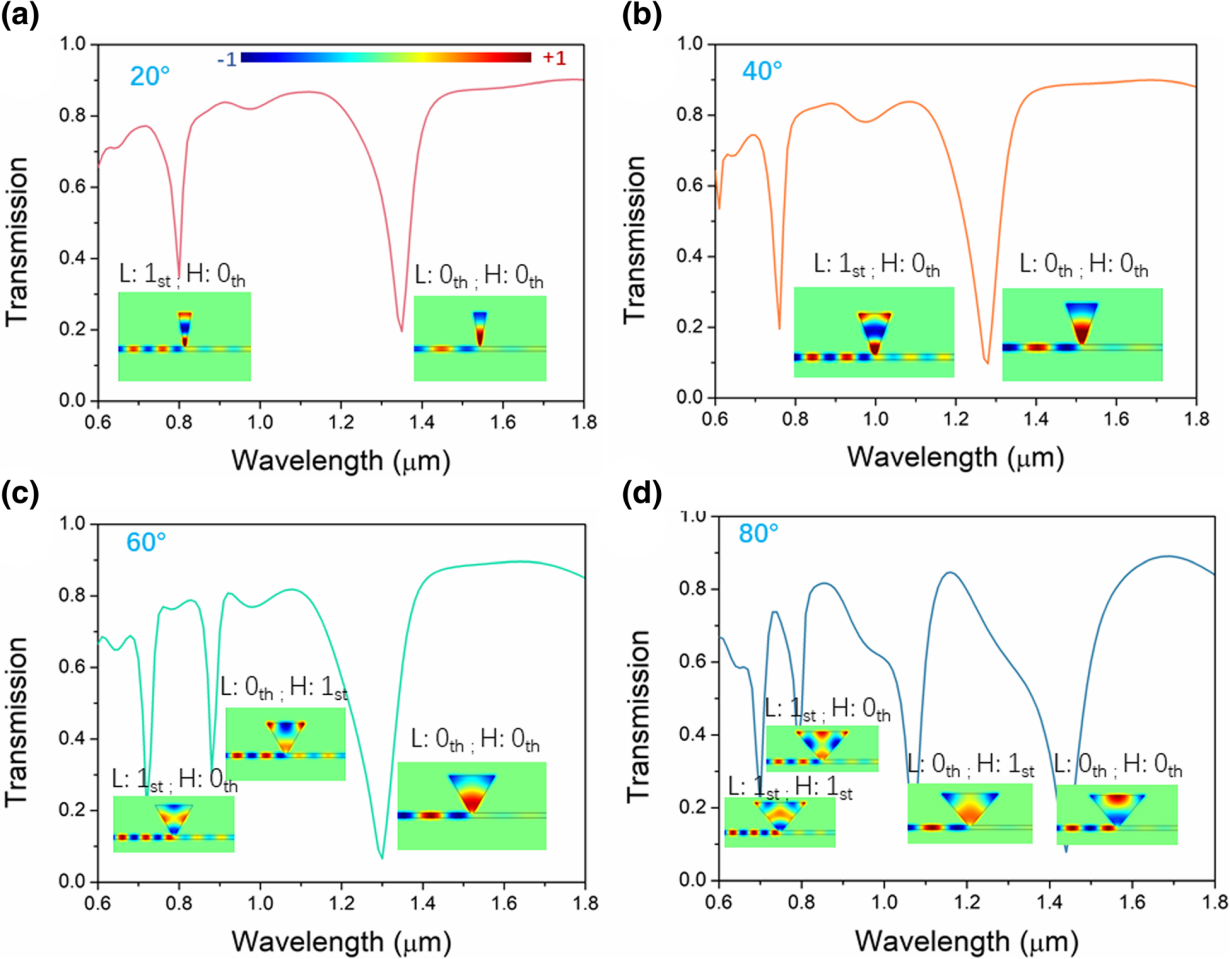
Transmission spectra of the single triangular resonator for angles of 20° (**a**), 40° (**b**), 60° (**c**), and 80° (**d**). Insets are magnetic field *H*_z_ corresponding to the resonant wavelengths

Unlike the normal rectangular resonators, the triangular resonators in the bowtie are determined by not only the side length, but also angles. So, we first investigate the impact of the angle connected to the bus waveguide on the transmission and resonant properties of the proposed structure with a single triangular resonator. The transmission spectra of single triangular resonator are shown in Fig. 2. All the heights of the resonator are fixed at 0.8 μm. The top angle of the triangular resonator is connected to the bus waveguide allowing electromagnetic energy side-coupled from the bus waveguide into the triangular resonator. Thus, deep transmission valleys appear on the spectra in Fig. 2. Those quantity, bandwidth, and valley wavelengths are determined by the structural parameters of the resonator. For the angle of 20°, there are two deep transmission valleys on the spectrum. The resonant valley at the longer wavelength is 0th order and 0th order in longitudinal and horizontal directions, respectively. With decreasing wavelength, the resonator height allows one more standing wave node, which is 1st order in the longitudinal direction. The situation for the angle of 40° is similar to that of 20°. As the angle increases, one more resonant valley emerges in the spectrum. The larger angle makes modal distribution split in a horizontal direction forming a high-order mode of 1st order in a horizontal direction. For a larger angle of 80°, the mode of L: 0th order splits in a horizontal direction forming L: 1st; H: 1st mode. Thus, the increasing angle results in both the shift of wavelength and the splitting of modal distribution in a horizontal direction forming high-order modes. The shift wavelength has no direct relations with the angle, because the variation of angle also alters the side length. So, to maintain the steady resonant properties, small angles are preferred.

**Fig. 3:**
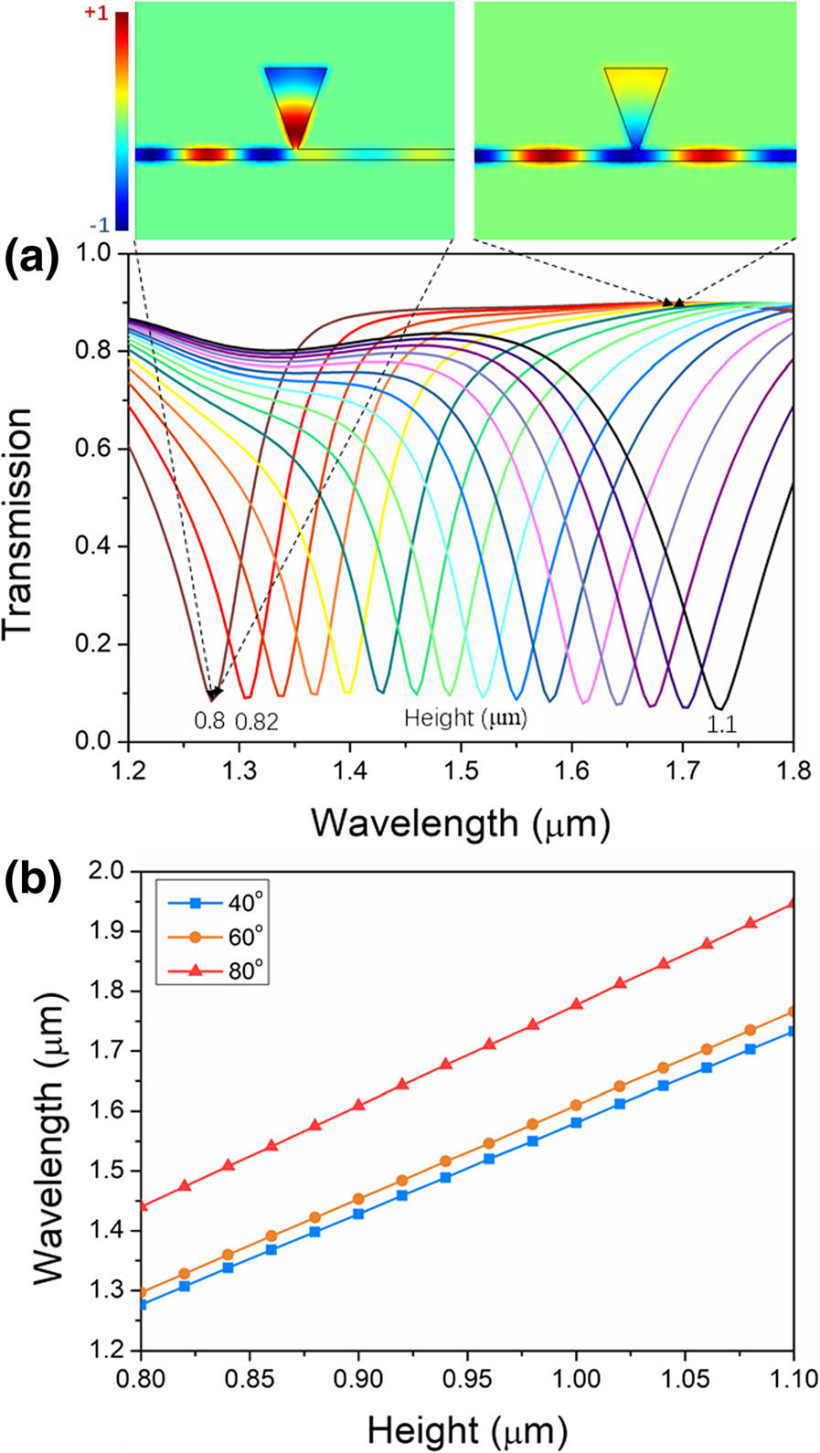
Transmission properties of the single triangular resonator**. a** Transmission spectra of the single triangular resonator for varying height. **b** Dependence of resonant wavelength on height for angles of 40°, 60°, and 80°. Insets are magnetic field *H*_z_ corresponding to the resonant and non-resonant wavelengths

The height of the resonator is the key parameter to the resonant properties. The transmission spectra of the device with a single triangular resonator for resonator height varying from 0.8 to 1.1 μm are shown in Fig. 3a. A cavity angle of 40° was selected during the simulation. Within the wavelength range from 1.2 to 1.8 μm, each of the spectra has a single dip, meaning the resonant valley. All the valley transmissivities are around 0.1. As the electromagnetic distribution of *H*_*z*_ at the resonant and non-resonant wavelengths shows in the insets of Fig. 3a, majority of the electromagnetic energy couples into the triangular resonator at the resonant wavelength, while most other wavelengths of the injected wideband light are transmitted through the bus waveguide. With the incremental height, the valley wavelength exhibits red shift behavior. As shown in Fig. 3b, the shifting wavelength is proportional to the height with excellent linearity. The shift of the resonant wavelength can be explained via the standing wave condition *Nλ*_*N*_ = 2*n*_*g*_*L*, *N* = (1, 2, 3…). For a specific *N*, the larger height of the triangular resonator causes the red shift of the resonant wavelength, while the shorter height causes the blue shift of resonant wavelength. For different angles, the relation between the resonant wavelength and height stays similar, which makes fabrication feasible without stringent requirements.

**Fig. 4:**
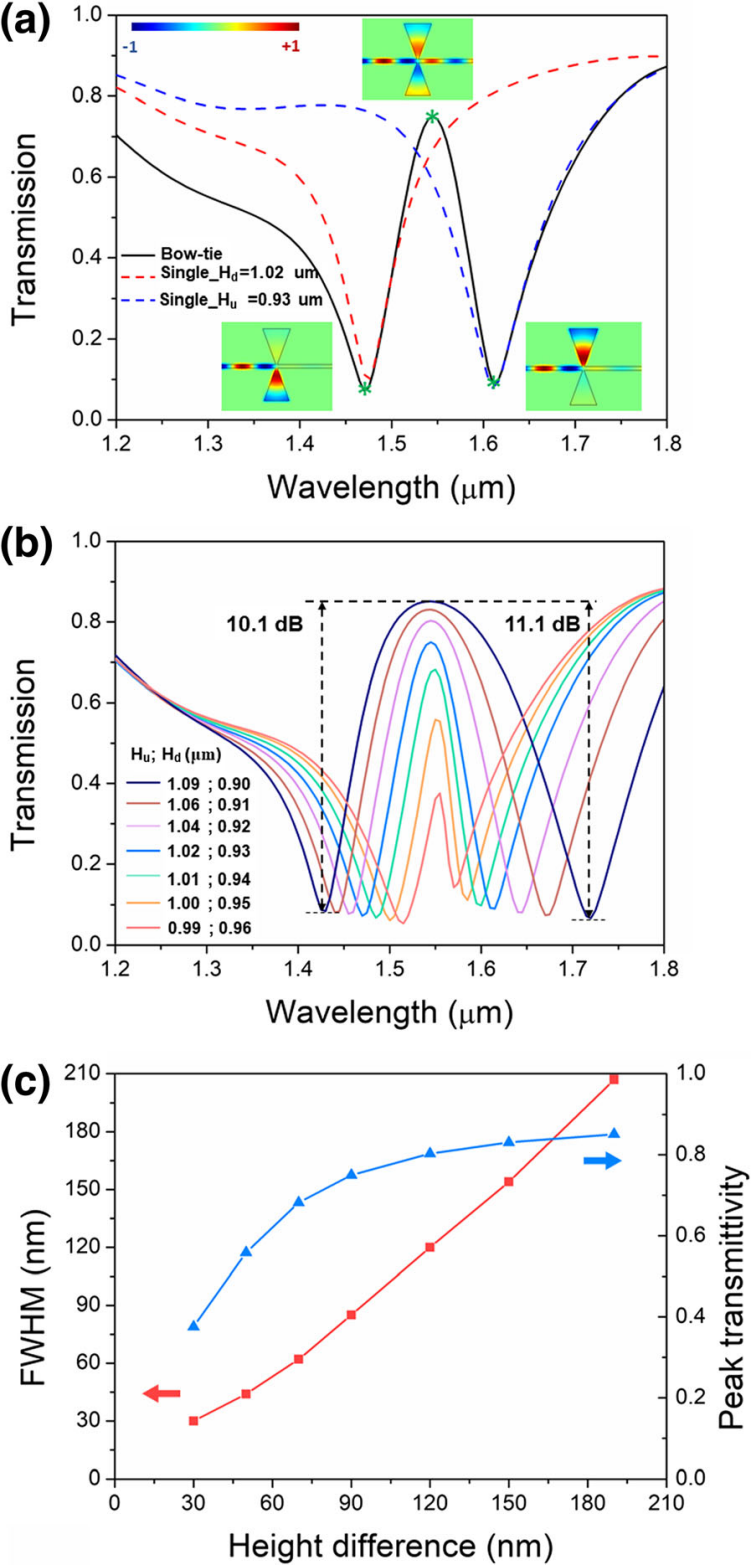
PIT transmission of the asymmetric bowtie structure. **a** PIT Transmission spectrum. **b** PIT transmission spectra for varying height difference. **c** FWHM and peak transmittivity as functions of height difference

In order to realize PIT transmission, strong coupling between double resonators with slightly detuned cavity length is required. The proposed asymmetric bowtie structure composed of triangular resonators with slightly detuned heights enables strong coupling between the resonators. By finely tuning the heights of the double triangular resonators, a transparent transmission peak will appear in the forbidden band of the single resonator. As shown in Fig. 4a, the angle of 20° was selected to maintain only one valley within the wavelength range and the heights were finely selected to make the PIT transmission band locate around 1.55 μm for applications in optical communications. The transmission spectrum of the single resonator with a height of 0.93 μm is depicted as the dashed red line. Its valley locates at 1.47 μm. To introduce structural difference along with valley difference, the single resonator with a height of 1.02 μm is employed to pair the previous resonator. The spectrum is depicted as the blue dashed line and its valley locates at 1.61 μm. Then, the electromagnetic energy inside the paired resonators strongly couples, forming a transmission spectrum with two deep valleys and one transparent peak, which is depicted as the black solid line. The transparent peak locates in the center between the two deep valleys, which was a forbidden band for single resonators. As the insets show, at the first valley, major electromagnetic energy couples into the resonator under the bus waveguide rather than the upper resonator. At the second valley, major electromagnetic energy couples into the upper resonator instead. These are very similar to those of single resonators. At the transparent peak, around 75% electromagnetic energy transmits through the bus waveguide, and only a small part of energy couples into the asymmetric bowtie resonators, forming a transparent band for the propagating electromagnetic energy. It is to be noted that PIT can be also obtained in asymmetric bowtie structure with different angles. However, the valley wavelength along with peak wavelength does not monotonically vary with the angle, leading to very difficult control of the transparent peak. Moreover, as mentioned in the above section, the resonator with larger angles gives rise to multi-mode resonance, which is detrimental to the control of the PIT effect. So, only the height difference-induced PIT is elaborated in this paper. The PIT effect in the proposed asymmetric bowtie structure is sensitive to the height. To keep the transparent peak at the optical communications wavelength, several sets of height values with height difference from 30 to 190 nm are selected to investigate the impact of height difference on the PIT effect. As shown in Fig. 4b, by finely selecting sets of resonator height values, the transparent peak can be kept at 1.55 μm. The maximum ratio of transparent peak to absorption valley can be more than 10 dB. The width and transmittivity both have a positive relation with height difference. In Fig. 4c, the full width at half maximum (FWHM) of the transparent band is proportional to the height difference with an approximately linear behavior, which is consistent with the behavior in Fig. 3b. Due to the existence of metallic dissipation, the totally transparent transmission of PIT effect is unpractical. The peak transmittivity first increases fast with the increasing height difference and then tends to be stable above 0.8.

**Fig. 5:**
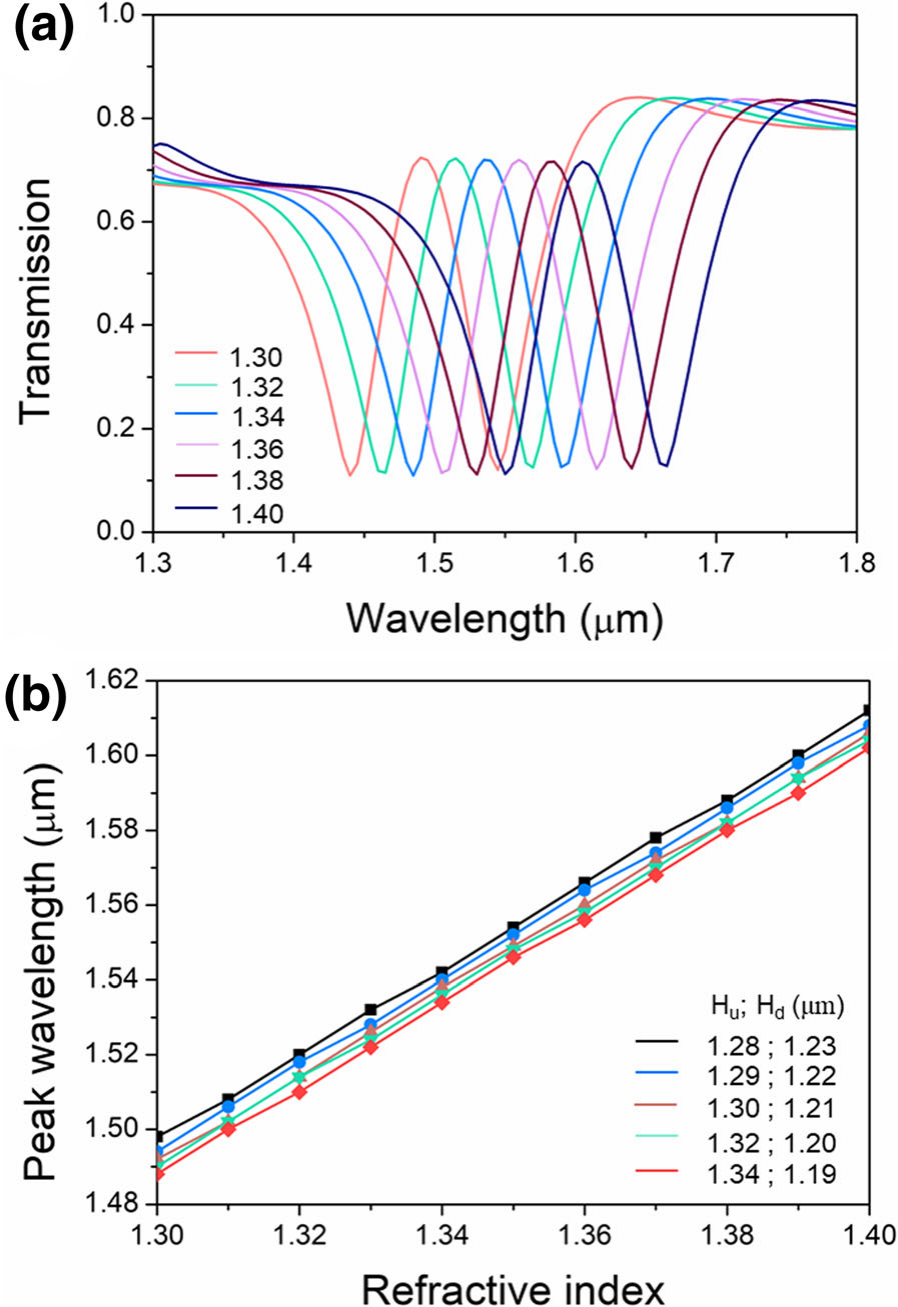
PIT-based sensing properties. **a** Transmission spectra of 90-nm height difference for refractive index varying from 80 to 120 nm. **b** Dependence of peak wavelength on refractive index for different height differences

As elaborated in the above sections, the valley and transparent peak are determined by the structural parameters and medium material inside the resonator and bus waveguide. So, the PIT-based sensing in the proposed asymmetric bowtie structure is feasible. Previously, the bus waveguide and resonators are filled by air, which means empty and can be employed as a container for liquid. In the simulation, the bus waveguide and resonators are filled by liquid. Its refractive index varies from 1.30 to 1.40, covering diverse common liquid of water, acetone, methyl alcohol, ethyl alcohol, propyl alcohol, glucose solution, etc. [26]. As shown in Fig. 5a, the transparent peak behaves red shift with the increasing refractive index of the liquid. Each peak can be obviously distinguished and the peak transmittivity nearly keeps stable. In Fig. 5b, the functions of peak wavelength as the refractive index for height differences of 50 nm, 70 nm, 90 nm, 120 nm, and 150 nm are directly proportional. The wavelength shift has excellent linearity. The calculated sensitivities for the height differences are all approximately equal to 1140 nm/RIU, and the corresponding sensing resolution is 8.8 × 10^−5^ RIU. So, the asymmetric bowtie PIT-based sensor has very high sensitivity and excellent immunity to fabrication deviation.

## Conclusions

We proposed an asymmetric bowtie structure to realize the PIT effect. Transmission properties of resonators with different structural parameters were numerically calculated using the finite element method. Through the strong coupling between the detuned triangular resonators, transparent transmission band can be obtained in the forbidden band of single resonators. With all three dimensions smaller than the free-space wavelength, the device has a simple and ultra-compact structure. The device also has excellent immunity to fabrication deviation making it easy to make without stringent requirements. Furthermore, PIT-based sensing property was demonstrated using the proposed asymmetric bowtie structure. The device can achieve a maximum sensitivity of 1140 nm/RIU; the corresponding sensing resolution is 8.8 × 10^−5^ RIU. The sensitivity has excellent linearity and consistency for varying height difference. Thus, our proposed asymmetric bowtie structure provides a new platform for on-chip EIT-like devices and refractive index sensors.

## Data Availability

The dataset is available without restriction.

## Abbreviations

EIT: Electromagnetically induced transparency
FWHM: Full width at half maximum
MIM: Metal-insulator-metal
PIT: Plasmon-induced transparency

## References

[1] Boller KJ, Imamoglu A, Harris SE (1991). Observation of electromagnetically induced transparency. Phys Rev Lett.

[2] Harris SE (1997). Electromagnetically induced transparency. Phys Today.

[3] Totsuka K, Kobayashi N, Tomita M (2007). Slow light in coupled-resonator-induced transparency. Phys Rev Lett.

[4] Huang Y, Min C, Veronis G (2011). Subwavelength slow-light waveguides based on a plasmonic analogue of electromagnetically induced transparency. Appl Phys Lett.

[5] Wu Y, Saldana J, Zhu Y (2003). Large enhancement of four-wave mixing by suppression of photon absorption from electromagnetically induced transparency. Phys Rev A.

[6] Liu C, Dutton Z, Behroozi CH, Hau LV (2001). Observation of coherent optical information storage in an atomic medium using halted light pulses. Nature.

[7] Liu N, Langguth L, Weiss T, Kastel J, Fleischhauer M, Pfau T, Giessen H (2009). Plasmonic analogue of electromagnetically induced transparency at the Drude damping limit. Nat Mat.

[8] Yanik MF, Suh W, Wang Z, Fan S (2004). Stopping light in a waveguide with all-optical analog of electromagnetically induced transparency. Phys Rev Lett.

[9] Xu Q, Sandhu S, Povinelli ML, Shakya J, Fan S, Lipson M (2006). Experimental realization of an on-chip all-optical analogue to electromagnetically induced transparency. Phys Rev Lett.

[10] Yang X, Hu X, Chai Z, Lu C, Yang H, Gong Q (2014). Tunable ultracompact chip-integrated multichannel filter based on plasmon-induced transparencies. Appl Phys Lett.

[11] Qiu P, Qiu W, Lin Z, Chen H, Ren J, Wang J, Kan Q, Pan J (2017). Dynamically tunable plasmon-induced transparency in on-chip graphene-based asymmetrical nanocavity-coupled waveguide system. Nanoscale Res Lett.

[12] Wang Y, Xie Y, Ye YC, Du Y, Liu B, Zheng W, Liu Y (2018). Exploring a novel approach to manipulating plasmon-induced transparency. Opt Commun.

[13] Certin AE, Artar A, Turkmen M, Yanik AA, Altug H (2011). Plasmon induced transparency in cascaded π-shaped metamaterials. Opt Express.

[14] Merbold H, Bitzer A, Feurer T (2011). Near-field investigation of induced transparency in similarly oriented double split-ring resonators. Opt Lett.

[15] Wang J, Yuan B, Fan C, He J, Ding P, Xue Q, Liang E (2013). A novel planar metamaterials design for electromagnetically induced transparency and slow light. Opt Express.

[16] Yan X, Wang T, Xiao S, Liu T, Hou H, Cheng L, Jiang X (2017). Dynamically controllable plasmon induced transparency based on hybrid metal-graphene metamaterials. Sci Rep.

[17] Han ZH, Bozhevolnyi SI (2011). Plasmon-induced transparency with detuned ultracompact Fabry-Perot resonators in integrated plasmonic devices. Opt Express.

[18] Gramotnev DK, Bozhevlnyi SI (2010). Plasmonic beyond the diffraction limit. Nat Photon.

[19] Wei W, Yan X, Shen B, Qin J, Zhang X (2018). Channel plasmon nanowire lasers with V-groove cavities. Nanoscale Res Lett.

[20] Polman A (2008). APPLIED PHYSICS Plasmonics applied. Science.

[21] William LB, Alain D, Thomas WE (2003). Surface plasmon subwavelength optics. Nature.

[22] Wei W, Zhang X, Ren X (2015). Plasmonic circular resonators for refractive index sensors and filters. Nanoscale Res Lett.

[23] Zhao H, Guang X, Huang J (2008). Novel optical directional coupler based on surface plasmon polaritons. Physica E.

[24] Zhang Q, Huang XG, Lin XS, Tao J, Jin XP (2009). A subwavelength coupler-type MIM optical filter. Opt. Experss.

[25] Johnson P, Christy R (1972). Optical constants of the noble metals. Phys Rev B.

[26] Rheims J, Köser J, Wriedt T (1997). Refractive-index measurements in the near-IR using an Abbe refractometer. Meas Sci Technol.

